# Extraordinary clinical benefit to sequential treatment with targeted therapy and immunotherapy of a BRAF V600E and PD-L1 positive metastatic lung adenocarcinoma

**DOI:** 10.1186/s40164-017-0089-y

**Published:** 2017-11-06

**Authors:** Shuyu D. Li, Annia Martial, Alexa B. Schrock, Jane J. Liu

**Affiliations:** 1 Department of Genetics and Genomic Sciences, Icahn Institute for Genomics and Multiscale Biology, Icahn School of Medicine at Mount Sinai, New York, NY 10029 USA; 2 Sema4, A Mount Sinai Venture, Stamford, CT 06902 USA; 3 Department of Internal Medicine, OSF St., Francis Medical Center, University of Illinois College of Medicine at Peoria, Peoria, IL 61605 USA; 4 Foundation Medicine, Inc., Cambridge, MA 02141 USA; 5 Illinois CancerCare, P.C., Peoria, IL 61615 USA

**Keywords:** Non-small cell lung cancer, BRAF V600E, PD-L1, Targeted therapy, Immunotherapy, Sequential treatment

## Abstract

**Background:**

The treatment algorithm for metastatic non-small cell lung cancers (NSCLCs) has been evolving rapidly due to the development of new therapeutic agents. Although guidelines are provided by National Comprehensive Cancer Network (NCCN) for treatment options according to biomarker testing results, sequentially applying the three main modalities (chemotherapy, targeted therapy and immunotherapy) remains an ad hoc practice in clinic. In light of recent FDA approval of dabrafenib and trametinib combination for metastatic NSCLCs with *BRAF* V600E mutation, one question arises due to insufficient clinical data is if the targeted therapy should be used before immunotherapy in patients with both *BRAF* V600E and PD-L1 expression.

**Case presentation:**

We present a case of 74-year-old female, former smoker with metastatic lung adenocarcinoma. The *BRAF* V600E mutation among other abnormalities was identified by comprehensive genomic profiling. The patient had an excellent 2-year response to the combination of pemetrexed and sorafenib. The patient was then treated with dabrafenib due to the presence of the *BRAF* V600E mutation and intolerance to cytotoxic chemotherapy. Not only the patient had an 18-month durable response to dabrafenib, she experienced outstanding quality of life with no serious adverse effects. At the time of symptomatic progression, the patient was then treated with two cycles of pembrolizumab based on her positive PD-L1 staining (90%). She had early response and came off pembrolizumab due to side effects. Seven months after initiation of pembrolizumab, the patient is off all the therapy and is currently asymptomatic. The patient is surviving with metastatic disease for over 7 years as of to date.

**Conclusions:**

By appropriately sequencing the three main modalities of systemic therapies, we are able to achieve long-term disease control with minimal side effects even in a geriatric patient with multiple comorbidities. We argue that it is reasonable to first use a BRAF inhibitor before considering immunotherapy for NSCLCs positive for both *BRAF* V600E and PD-L1.

**Electronic supplementary material:**

The online version of this article (10.1186/s40164-017-0089-y) contains supplementary material, which is available to authorized users.

## Background

The treatment paradigm for metastatic non-small cell lung cancers (NSCLCs) has been evolving rapidly due to new therapeutic options [1]. In metastatic, non-small cell, non-squamous lung cancer patients, three groups can be defined based on tumor molecular testing results, each paired with a specific first-line systemic therapy of proven clinical benefit. Patients in the first group are positive for sensitizing *EGFR* mutations, *ALK* or *ROS1* rearrangement with the matched targeted tyrosine kinase inhibitors (TKIs) as the first-line treatment. In the second group, patients are PD-L1 immunohistochemistry positive (≥ 50%) and *EGFR*, *ALK*, *ROS1* negative, and single agent pembrolizumab is a FDA-approved first-line therapy. Patients in the third group are *EGFR*, *ALK*, *ROS1*, and PD-L1 negative, paired with systemic chemotherapy plus or minus pembrolizumab as the first-line option. Significant progress has also been made to develop predictive biomarkers for PD-1/PD-L1 immune checkpoint blockade therapy [2, 3].

In addition to *EGFR*, *ALK* and *ROS1*, emerging evidences have demonstrated clinical benefit to therapies against BRAF [4–7], MET [8–10], RET [11, 12] or HER2 [13, 14] in NSCLCs harboring activating mutations. Most notably, FDA approved dabrafenib and trametinib combination for metastatic NSCLCs with *BRAF* V600E mutation on June 22, 2017 (https://www.fda.gov/drugs/informationondrugs/approveddrugs/ucm564331.htm). In light of this recent regulatory approval, one question arises due to insufficient clinical data is if the targeted therapy should be used before immunotherapy in patients with both *BRAF* V600E and PD-L1 expression.

## Case presentation

**Fig. 1:**
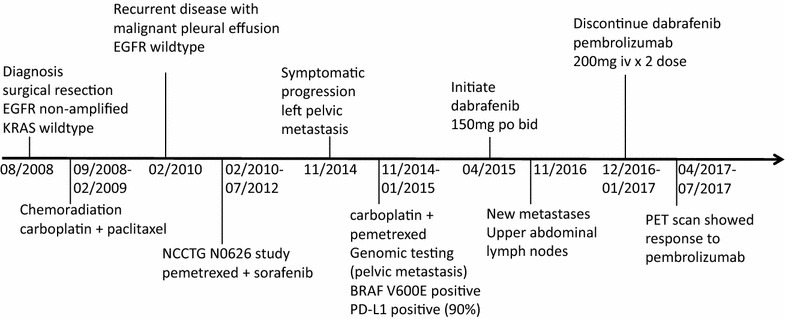
Oncology history of the patient

**Fig. 2:**
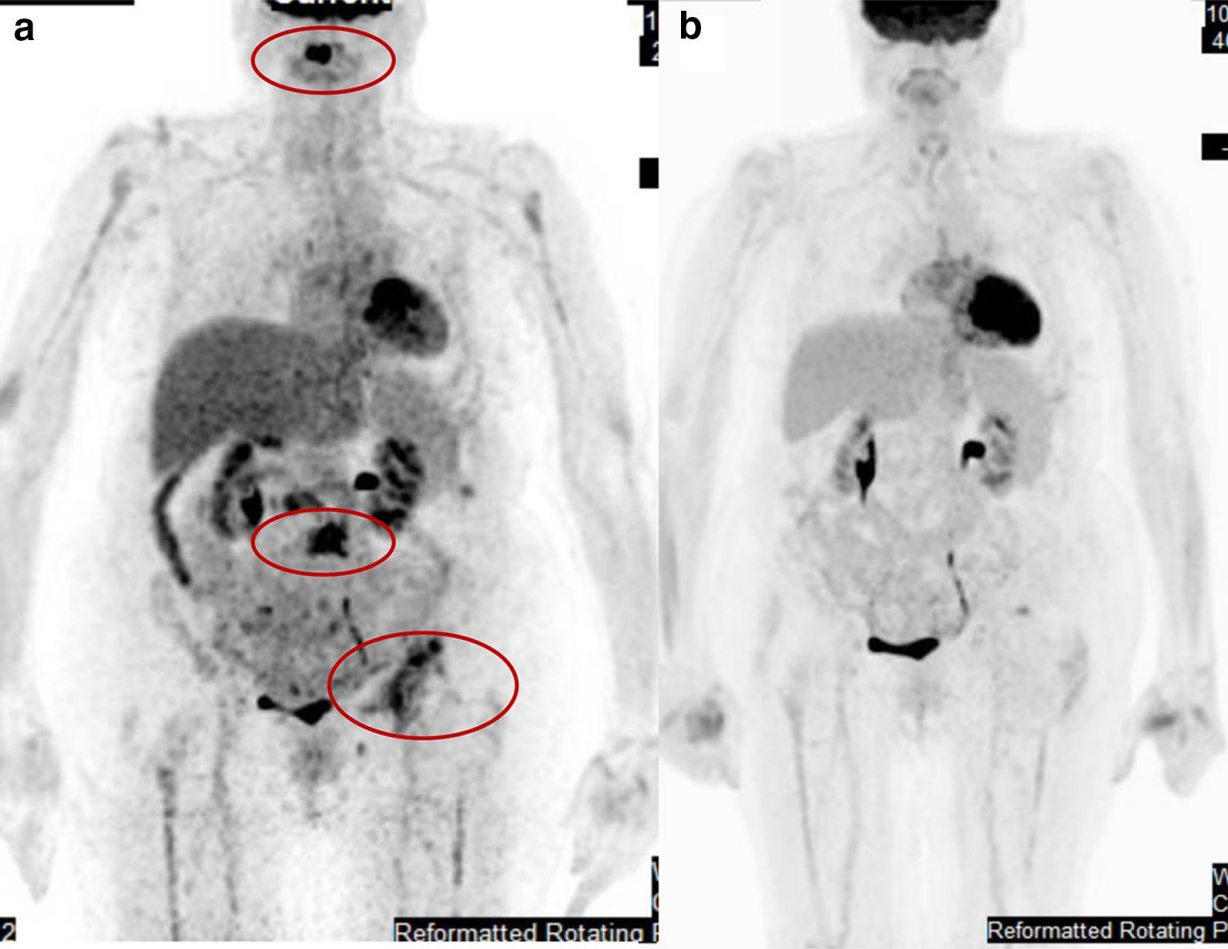
**a** PET scan of the patient before initiation of dabrafenib reveals metastatic disease to the left iliac bone, C2 and L3-4 vertebral bodies. The C2 lesion’s SUV max was 7; the lesion at L3 had a SUV max of 7.1; the left acetabulum lesion’s SUV max was 5.1 prior to starting dabrafenib. **b** After 4 months of dabrafenib therapy, near complete resolution of PET activity in the areas of bone metastases was demonstrated without any new site of disease. Upon the best response to dabrafenib achieved, the metabolic activity resolved at C2 and L3 lesions. The left acetabulum lesion only had a very small focus of residual uptake that the max SUV was not measured

A 74-year-old female, former smoker had resected stage III lung adenocarcinoma and was treated with adjuvant concurrent chemoradiation with carboplatin and paclitaxel in 2008 (Fig. 1). The patient’s surgical resection specimen was tested for *EGFR* amplification by FISH (ARUP Laboratories) and *KRAS* mutation analysis (GenPath Diagnostics), and the results indicated *EGFR* was non-amplified and KRAS was wild type at codons 12, 13, and 61. Her medical history includes hypertension, hyperlipidemia, GERD (gastroesophageal reflux disease), SVT (supraventricular tachycardia), chronic kidney disease and osteoporosis. The patient developed metastatic recurrent lung cancer with malignant pleural effusion in 2010. The *EGFR* mutation analysis by real-time PCR (Clarient Diagnostic Services) was done on the pleural effusion specimen and none of the 29 known mutations, deletions and insertions found in exons 18–21 of the EGFR tyrosine kinase domain was detected. The patient was then treated with pemetrexed and sorafenib on trial (NCCTG N0626 study, http://ascopubs.org/doi/abs/10.1200/jco.2011.29.15_suppl.7513) with a durable response for more than 2 years (Fig. 1). The treatment was stopped in 2012 due to intolerance. Afterwards, the patient was on observation for 2 years until she developed symptomatic progression with extensive bony metastasis in 2014 (Figs. 1, 2a). Her left pelvic metastasis biopsy specimen was used for genomic profiling and PD-L1 staining (see below). She was treated with palliative radiation, followed by carboplatin and pemetrexed. Cytotoxic chemotherapy was discontinued after 2 months due to profound toxicities which required hospitalization, despite of dose reductions (Fig. 1).

To explore additional therapeutic options, we then performed comprehensive genomic profiling (CGP) using the FoundationOne^**®**^ panel (http://foundationone.com/). The CGP identified the *BRAF* V600E mutation as well as inactivating mutations in tumor suppressors including *ATM*. In addition, the tumor mutation burden was low—five per megabase and the tumor was microsatellite stable (MSS). The full report of the CGP is provided as Additional file 1. Based on this genetic profile, the patient was started on dabrafenib [7] in April 2015 (Fig. 1). Dabrafenib was used at 150 mg PO BID throughout the treatment course. She had excellent clinical and radiographic responses (Fig. 2b). Her performance status drastically improved. Her only noticeable side effect was hypokalemia that was managed with oral and IV potassium replacement. The patient developed increase of metabolic activity from two disease sites on PET scan suggestive of disease progression without clinical symptoms 7 months after initiation of dabrafenib. Based on the phase II trial [6] reported in 2015 ASCO annual meeting demonstrating activity of dabrafenib and trametinib combination in *BRAF* mutated lung cancers, our patient was offered the addition of trametinib. However, she did not tolerate the combination and stopped trametinib after 1 week. The patient was asymptomatic from her metastatic lung cancer until the 19th month into the dabrafenib therapy, when she developed productive cough and restaging scan revealed new hypermetabolic upper abdominal lymph node metastases at the gastrohepatic ligament, precaval and peripancreatic retroperitoneum (Fig. 1).

The patient was discontinued from dabrafenib and started on pembrolizumab based on her positive PD-L1 staining (90%) in December 2016 (Fig. 1). The treatment was complicated by immune mediated colitis and pneumonitis which promptly responded to systemic steroids. The dose and duration of steroids used for treating pneumonitis are as follows: prednisone 40 mg daily for 1 weeks, followed by 20 mg daily for 5 days, 10 mg daily for 5 days, 5 mg daily for 5 days, then off. Her colitis was successfully treated in the similar fashion. A repeat CT scan 12 days after initiation of pembrolizumab was done for the work-up of abdominal pain, confirmed colitis, but also demonstrated decreased size of the gastric hepatic ligament node and resolution of the peripancreatic nodule, consistent with early response. She was able to stop steroid and became asymptomatic from her disease and prior treatment effects in March 2017. As of July 2017, the patient has no signs of disease progression after only two doses of pembrolizumab (200 mg IV) 7 weeks apart without additional therapy (Fig. 1). The patient has not had any hospitalization after the initiation of dabrafenib. It is noted that a recent pooled analysis of advanced melanoma (http://ascopubs.org/doi/abs/10.1200/JCO.2017.73.2289) also showed the patients who discontinued PD-1 checkpoint blockade antibodies continue to benefit from the treatment.

## Discussion and conclusions

We present a case of *BRAF* V600E positive and PD-L1 positive metastatic lung adenocarcinoma. The patient exhibited excellent response for more than 18 months to single agent dabrafenib. Although serious adverse events (AEs) were observed in 42% of patients in the single-arm, phase II trial of dabrafenib [7] with skin toxicities as the most frequent grade 3 or worse AEs, the patient in our case only displayed manageable hypokalemia with no skin toxicity.

Prior to dabrafenib, the patient also demonstrated 2-year response to a pemetrexed and sorafenib based regimen. After the treatment was stopped, the patient had another 2 years of stable disease before disease progression. This excellent response could be partially due to the presence of the *BRAF* V600E mutation. Although previous phase III studies of sorafenib in NSCLC failed to meet primary end points [15], *BRAF* mutation status was neither used in trial design nor analyzed retrospectively as a biomarker. Our results suggest *BRAF* activating mutations could be a patient stratification marker in NSCLC trials incorporating sorafenib. Notably, a recent case report demonstrated efficacy of sorafenib in a NSCLC harboring activating *BRAF* G469V mutation, but no response in synchronous *BRAF* wild type-hepatocellular carcinoma [16].

As our patient was positive for PD-L1 (90%), pembrolizumab treatment was initiated and the patient demonstrated response with stable disease radiographically. Since the tumor harbors an inactivating mutation in *ATM*, the response to anti-PD1 therapy is also consistent with previous studies that DNA repair deficiency predicts immunotherapy response [17, 18]. Interestingly, our patient has a low tumor mutation burden (TMB). The presence of the *BRAF* V600E mutation, high PD-L1 expression, and response to pembrolizumab in our case supports a recent preliminary report (http://www.abstractsonline.com/pp8/#!/4292/presentation/1306) that TMB-low/PD-L1-high NSCLCs are enriched for *BRAF* mutations suggesting *BRAF* alterations in this group may trigger immune responses moderated by PD-L1 expression.

Systemic chemotherapy in advanced NSCLC results in a median overall survival (OS) of only 8 to 12 months and a median progression-free survival (PFS) of 5 to 6 months [19–21]. First line targeted TKIs significantly improved outcome: 10–14 months of PFS and 20–32 months of OS for EGFR-TKIs [22–27], and 15.3 months of PFS and 36.8 months of OS (http://abstracts.asco.org/199/AbstView_199_183873.html) for ALK-TKIs. It is remarkable that our patient is surviving with metastatic lung cancer for over 7 years as of to date. From this case, we argue that it is reasonable to consider a BRAF inhibitor before utilizing immunotherapy in patients with *BRAF* V600E-positive and PD-L1 positive metastatic NSCLC. Our patient had excellent quality of life and more than 18 months of disease control on a BRAF inhibitor. She has been free of hospitalization and emergency room visit since the initiation of dabrafenib. This demonstrated a successful case of transitioning advanced lung cancer into a chronic disease. The advent of targeted therapy and immunotherapy made it possible to achieve long-term disease control with minimal side effects even in a geriatric patient with multiple comorbidities. Appropriately sequencing the three main modalities of systemic therapies (cytotoxic chemotherapy, targeted therapy and immunotherapy) to achieve long-term disease control and minimize side effect is the ultimate goal in modern age of lung cancer care, and this case report provides practicing oncologists a valuable reference.

We should also point out that in addition to considering each therapeutic modality individually, there are significant efforts to explore combination of immunotherapy plus standard chemotherapy or combination of immunotherapy plus radiotherapy [28–30]. For example, in a phase II study of pembrolizumab in combination with carboplatin and pemetrexed in chemotherapy-naïve, advanced non-squamous NSCLCs, the pembrolizumab plus chemotherapy group achieved an objective response rate of 55% in comparison to 29% in the chemotherapy alone group while the incidence of grade 3 or worse treatment-related adverse events was similar between the two groups [29]. Progression-free survival was also significantly longer with pembrolizumab plus chemotherapy compared with chemotherapy alone [29]. In a phase I study, radiation therapy in combination with pembrolizumab is evaluated (NCT02318771), and immune biomarkers for treatment failure in a specific case was reported [28]. Collectively, these studies may represent a future direction to develop more effective treatment options for NSCLCs.

We recognize the limitation of a single case report and several factors one should take into considerations. Although our case suggests sequencing BRAF-TKIs followed by pembrolizumab might be considered for advanced NSCLCs positive for both *BRAF* V600E and PD-L1, this is only a single case for which the clinical decision was made based on this particular patient’s prior treatment history, response and side effects. Additional clinical studies are needed to provide more clinical evidences. In metastatic NSCLCs driven by other oncogenes with available matched TKIs, the patients often do not benefit from immunotherapy. For example, a meta-analysis of randomized trials comparing immune checkpoint inhibitors against chemotherapy as second line therapy in EGFR-mutant advanced NSCLC concluded immune checkpoint inhibitors do not improve OS over that with docetaxel [31]. A recent study presented in 2017 ASCO annual meeting also shows that NSCLCs harboring *MET* exon-14 alterations responded poorly to immunotherapy, even in PD-L1 positive patients (http://abstracts.asco.org/199/AbstView_199_189471.html). Moreover, a small percentage of patients develop hyper-progressive disease (HPD) after treatment with immune checkpoint inhibitors, and this hyper-progression appears to be associated with MDM2 amplification or EGFR alterations in a recent study [32]. We also need to consider AEs when TKIs and immunotherapy are administered concurrently or sequentially. For example, in EGFR-mutant NSCLCs, nivolumab and erlotinib combination was associated with 19% of grade 3 toxicities, and osimertinib and durvalumab combination resulted in significantly elevated incidence of interstitial lung disease [33]. In our case, the patient experienced colitis and pneumonitis upon pembrolizumab treatment, although they were mitigated through systemic steroids. Nevertheless, extra caution should be exercised to ensure sequential or concurrent treatment with targeted TKIs and immunotherapy are applied safely.

In conclusion, here we present a unique case of NSCLC where we transitioned an advanced lung cancer into a chronic disease in a geriatric patient. Sequential treatment with BRAF-TKIs and immunotherapy could provide significant clinical benefit to metastatic lung adenocarcinomas positive for both *BRAF* V600E and PD-L1.

## Additional file

**Additional file 1.** Full report of comprehensive genomic profiling (CGP) based on FoundationOne^®^ panel.

## Abbreviations

NSCLC: non-small cell lung cancer
TKI: tyrosine kinase inhibitor
CGP: comprehensive genomic profiling
AE: adverse event
OS: overall survival
PFS: progression-free survival
TMB: tumor mutation burden
