# Supplementary material for: Extraordinary clinical benefit to sequential treatment with targeted therapy and immunotherapy of a BRAF V600E and PD-L1 positive metastatic lung adenocarcinoma
Source: Exp Hematol Oncol. 2017 Nov 6;6:29. doi: 10.1186/s40164-017-0089-y (PMC5674807; doi:10.1186/s40164-017-0089-y)
Supplement: Supplementary file 1 — Additional file 1. Full report of comprehensive genomic profiling (CGP) based on FoundationOne® panel. [file 40164_2017_89_MOESM1_ESM.pdf]

FOUNDATIONONE™

Patient Name	Report Date	Tumor Type
[illegible]n	05 January 2015	Lung adenocarcinoma

**Date of Birth**		**Medical Facility**	Illinois Cancer Care	**Specimen Received**	24 December 2014
**Sex**	Female	**Ordering Physician**	Liu, Jane	**Specimen Site**	Pelvis
**FMI Case #**		**Additional Recipient**	Not Given	**Date of Collection**	24 November 2014
**Medical Record #**	Not Given	**Medical Facility ID #**	201075	**Specimen Type**	Block
**Specimen ID**	[redacted]	**Pathologist**	Not Provided		

ABOUT THE TEST:
FoundationOne® is a next-generation sequencing (NGS) based assay that identifies genomic alterations within hundreds of cancer-related genes.

Note: This is a QUALIFIED report. This specimen failed to meet minimum performance standard following sequencing. We can confirm the presence of the genomic alterations detailed in this report, but the data obtained were insufficient for comprehensive detection of genomic alterations.

PATIENT RESULTS

- 4 genomic alterations
- 4 therapies associated with potential clinical benefit
- 0 therapies associated with lack of response
- 8 clinical trials

TUMOR TYPE: LUNG ADENOCARCINOMA

Genomic Alterations Identified†

BRAF V600E
ATM Y264fs*12
ARID2 splice site 5225_5271+38del85
SETD2 T1054fs*5

Additional Disease-relevant Genes with No Reportable Alterations Identified†

RET
ALK
KRAS
ERBB2
MET
EGFR

†For a complete list of the genes assayed and performance specifications, please refer to the Appendix
⌘See Appendix for details

THERAPEUTIC IMPLICATIONS

Genomic Alterations Detected	FDA Approved Therapies (in patient's tumor type)	FDA Approved Therapies (in another tumor type)	Potential Clinical Trials
**BRAF** V600E	None	Dabrafenib<br>Regorafenib<br>Trametinib<br>Vemurafenib	Yes, see clinical trials section
**ATM** Y264fs*12	None	None	Yes, see clinical trials section
**ARID2** splice site 5225_5271+38del85	None	None	None

For additional information please log on to the Interactive Cancer Explorer™
To set up your Interactive Cancer Explorer account, contact your sales representative or call (888) 988-3639.

**SETD2** T1054fs*5	None	None	None

Note: Genomic alterations detected may be associated with activity of certain FDA approved drugs; however, the agents listed in this report may have varied clinical evidence in the patient's tumor type. Neither the therapeutic agents nor the trials identified are ranked in order of potential or predicted efficacy for this patient, nor are they ranked in order of level of evidence for this patient's tumor type.

GENOMIC ALTERATIONS

GENE ALTERATION	INTERPRETATION
**BRAF** V600E	BRAF encodes a member of the RAF family of protein kinases, which includes ARAF, BRAF and CRAF. RAS promotes BRAF activation, which leads to the activation of the MAPK (RAF-MEK-ERK) signaling cascade[1]. BRAF V600E, which is located in the kinase domain, leads to constitutive BRAF activation and downstream MEK-ERK signaling, and promotes oncogenic transformation[2,3]. BRAF mutations have been reported in up to 20% of all cancers, with the majority of mutations occurring at the V600 position[3,4]. BRAF mutations have been reported in up to 4% of non-small-cell lung cancer (NSCLC) patients in various studies (An et al., 2013; ASCO Abstract 8101)[5,6,7,8], with a large-scale meta-analysis putting the frequency at 3%[9]. BRAF mutations are significantly more prevalent in lung adenocarcinoma than non-adenocarcinoma NSCLC[6,9]. Studies have reported a lack of association between BRAF mutation and tumor stage or prognosis (An et al., 2013; ASCO Abstract 8101)[9]. BRAF mutations can co-occur with alterations in other known oncogenic drivers of NSCLC, including EGFR, KRAS, and ALK (An et al., 2013; ASCO Abstract 8101)[6,8]. BRAF V600E activates MEK-ERK signaling and is associated with sensitivity to BRAF V600 mutant-specific inhibitors (such as vemurafenib and dabrafenib)[10,11], multikinase inhibitors that have activity against BRAF (such as regorafenib)[12], MEK inhibitors (such as trametinib)[13], and ERK inhibitors[14]. Vemurafenib, dabrafenib, and trametinib are FDA approved for the treatment of melanoma with BRAF V600 mutations[10,11,13]. Regorafenib is FDA approved in metastatic colorectal cancer and advanced gastrointestinal stromal tumors (GISTs)[15,16] and was reported to have clinical activity in one colorectal cancer patient with the BRAF V600E mutation[12]. Vemurafenib and dabrafenib have elicited clinical responses in lung adenocarcinoma patients with the BRAF V600E mutation (Planchard et al., 2013; ASCO Abstract 8009)[17,18,19]. Additionally, inhibition of HSP90 leads to the degradation of oncogenic proteins, such as BRAF, which suggests that HSP90 inhibitors may be effective in malignancies with activating BRAF mutations; indeed, combinations of BRAF or MEK inhibitors with other therapies including HSP90 inhibitors, immunotherapies, PI3K/AKT inhibitors, ERK inhibitors, and/or CDK4/6 inhibitors are in clinical trials[20,21,22].
**ATM** Y264fs*12	ATM encodes the serine/threonine protein kinase 'ataxia telangiectasia mutated', a member of the PI3K/PI4K family of kinases. ATM plays a key role in sensing double-strand DNA breaks and activating cellular checkpoint pathways, arresting the cell cycle when DNA damage is present[23]. Cells that are deficient for ATM progress through the cell cycle even in the presence of DNA damage, resulting in the accumulation of DNA errors and genomic instability that can lead to cancer[23]. ATM mutations that disrupt the protein kinase domain (amino acids 2712-2962) or the FATC domain (amino acids 3024-3056), such as observed here, are predicted to result in loss of function[24]. ATM mutations have been reported in 6-9% of lung adenocarcinomas analyzed (cBioPortal, COSMIC, Sep 2014)[25]. There are currently no approved therapies directly targeting ATM mutation or loss. However, preclinical evidence suggests that ATM-deficient tumors may be sensitive to PARP inhibitors[26,27]. PARP inhibitors have been investigated both alone and in combination therapeutic strategies in several cancer types in clinical studies and are currently under investigation in clinical trials for advanced solid tumors[28,29]. A preclinical study suggested that functional loss of ATM leads to dependence on DNA-PKcs (DNA-dependent protein kinase catalytic subunit); DNA-PKcs inhibitors were shown to promote apoptosis in ATM-deficient cells, and to be active in vivo against lymphomas lacking active ATM, suggesting a promising therapeutic strategy for tumors with inactivating ATM mutations[30].
**ARID2** splice site 5225_5271+38del85	ARID2 encodes a subunit of the chromatin remodeling PBAF protein complex, which plays a role in ligand-dependent activation of gene expression by nuclear receptors[31]. Frequent mutation of ARID2 has been observed in hepatitis C-associated hepatocellular carcinoma (14-18% of tumors)[32,33], and mutations have also been reported in microsatellite unstable colorectal cancer (13%)[34], pancreatic cancer[35,36], melanoma (7%)[37], and non-small-cell lung cancer (5%)[38]. The majority of the ARID2 mutations identified are truncations, splice site defects or inactivating rearrangements, suggesting that ARID2 functions as a tumor suppressor in these contexts. ARID2 mutations have been detected in less than 0.5% of all hematopoietic and lymphoid samples in COSMIC (2014). Currently, there are no targeted therapies to address inactivation of ARID2.

**SETD2**<br>T1054fs*5	SETD2 encodes a histone lysine-36 methyltransferase[39] that preferentially interacts with the expanded N-terminal polyglutamine tracts present in mutant huntingtin, implicating it in the pathogenesis of Huntington disease[40]. SETD2 mRNA expression has been observed to be consistently reduced in breast tumors relative to adjacent non-tumor tissue, suggesting a potential tumor suppressor role[41]. Somatic inactivating alterations of SETD2 are documented to occur at low frequency in a number of solid tumors, most commonly in renal carcinoma[42]. Currently, there are no targeted therapies available to address genomic alterations in SETD2.

THERAPIES

There are no therapies FDA approved in this patient's tumor type that are specific to the reported genomic alterations.

ADDITIONAL THERAPIES – FDA APPROVED IN OTHER TUMOR TYPES

THERAPY	RATIONALE
Dabrafenib	Dabrafenib is a BRAF inhibitor that has been approved by the FDA for use in unresectable or metastatic melanoma with a BRAF V600E mutation, based on Phase 3 clinical trials reporting significant improvements in progression-free survival, as compared with dacarbazine; BRAF V600E mutations have been shown to predict sensitivity to dabrafenib in melanoma[11]. A clinical trial of dabrafenib in BRAF V600E mutation-positive NSCLC patients has reported partial responses (PR) in 7/13 patients and stable disease (SD) in 1/13 patients, with grade 3 or higher adverse events (AE) seen in 6 patients (Planchard et al., 2013; ASCO Abstract 8009). A case study has reported a PR in a BRAF V600E mutant lung adenocarcinoma patient treated with dabrafenib, whose cancer acquired resistance in association with a secondary KRAS mutation[19]. Dabrafenib can induce adverse effects such as the development of cutaneous squamous cell carcinomas and keratoacanthomas caused by inactivation of wild-type BRAF and leading to paradoxical activation of the MAPK pathway[11,43]. Melanoma patients with BRAF V600E or V600K mutation treated with a combination of dabrafenib with the MEK inhibitor trametinib experienced significantly lower rates of cutaneous squamous cell carcinoma[44].
Regorafenib	Regorafenib is a multikinase inhibitor that inhibits multiple kinases, including RET, VEGFRs, PDGFRs, Kit, FGFR1, FGFR2, and RAF family proteins[45]. Regorafenib is FDA approved for the treatment of metastatic colorectal cancer or advanced gastrointestinal tumors (GIST)[15,16,46]. BRAF activating mutations may predict sensitivity to RAF inhibitors such as regorafenib. A case study of a heavily pretreated metastatic CRC patient whose tumor harbored a BRAF V600E mutation reported clinical activity of regorafenib as a single agent and in combination with panitumumab[12]. Regorafenib use has been linked to the development of intestinal perforation in a CRC patient and a GIST patient[47].
Trametinib	Trametinib is a MEK inhibitor that is FDA approved as both a single agent and in combination with dabrafenib for the treatment of unresectable or metastatic melanoma with BRAF V600E or V600K mutations. Melanoma patients with BRAF V600E or V600K mutation treated with a combination of dabrafenib and trametinib experienced significantly improved progression free survival (PFS) (9.4 months vs 5.8 months in Phase 1-2, 9.3 months vs 8.8 months in Phase 3 trials) and response rate (RR) (76% vs 54% in Phase 1-2, 67% vs 51% in Phase 3) compared to dabrafenib alone; this combination resulted in significantly lower rates of cutaneous squamous cell carcinoma and regression of established BRAF inhibitor-induced skin lesions[44,48,49]. Trametinib therapy for the treatment of non-small cell lung cancer (NSCLC) has primarily been studied in the context of KRAS mutation status, and responses associated with alterations in other genes have not been widely described (PubMed, Nov 2014). Phase 1 and 2 monotherapy trials of MEK inhibitors such as trametinib and RO4987655 have shown low response rates in patients with NSCLC, irrespective of KRAS mutation status, and no improvement in progression-free survival compared to docetaxel (Blumenschein et al., 2013; ASCO Abstract 8029)[50,51]. However, Phase 1 and 2 trials of MEK inhibitors in combination with docetaxel or pemetrexed in NSCLC have shown improved clinical activity and patient survival compared to chemotherapeutics alone, although no association was observed between response and KRAS mutation status (Kelly et al., 2013; ASCO Abstract 8027, Gandara et al., 2013; ASCO Abstract 8028)[52]. In contrast, Phase 2 trials of the MEK inhibitor selumetinib in combination with erlotinib showed no responses in patients with NSCLC, irrespective of KRAS mutation status, and increased toxicity of the combination compared to either erlotinib or selumetinib alone (Carter et al., 2013; ASCO Abstract 8026). Preclinical and early clinical studies have shown synergistic anti-tumorigenic effects when the combination of MEK and PI3K inhibitors was used to treat KRAS-driven NSCLC (Banerji et al., 2014; ASCO Abstract e13559)[53,54]. However, a Phase 1b trial of a combination of trametinib and the mTOR inhibitor everolimus in patients with solid tumors reported frequent adverse events and the study was unable to identify a recommended Phase 2 dose and schedule for the combination[55].

For additional information please log on to the Interactive Cancer Explorer™

To set up your Interactive Cancer Explorer account, contact your sales representative or call (888) 988-3639.

Vemurafenib	Vemurafenib is a BRAF V600-mutant selective inhibitor that is FDA approved as a single agent for the treatment of unresectable or metastatic melanoma with the BRAF V600E mutation. Metastatic melanoma patients with the BRAF V600E mutation treated with vemurafenib had significantly improved overall survival (OS), progression-free survival (PFS), and response rate (RR) compared with patients treated with dacarbazine[10]. Lung adenocarcinoma patients with the BRAF V600E mutation have been reported to respond to vemurafenib, including one report of a complete response[17,18]. Vemurafenib treatment has been linked to pulmonary injury[56]. Additionally, vemurafenib can induce adverse effects such as the development of cutaneous squamous cell carcinomas, keratoacanthomas, and new primary melanomas caused by inactivation of wild-type BRAF and leading to paradoxical activation of the MAPK pathway[10,43]. In a phase 1 trial, melanoma patients with the BRAF V600E mutation treated with a combination of vemurafenib with the MEK inhibitor cobimetinib experienced lower rates of cutaneous squamous cell carcinoma[57].

Genomic alterations detected may be associated with activity of certain FDA approved drugs, however the agents listed in this report may have little or no evidence in the patient's tumor type

CLINICAL TRIALS TO CONSIDER

IMPORTANT: While every effort is made to ensure the accuracy of the information contained below, the information available in the public domain is continuously updated and should be investigated by the physician or research staff. This is not meant to be a complete list of available trials. In order to conduct a more thorough search, please go to www.clinicaltrials.gov and use the search terms provided below. For more information about a specific clinical trial, type the NCT ID of the trial indicated below into the search bar.

GENE	RATIONALE FOR POTENTIAL CLINICAL TRIALS
**BRAF** V600E	BRAF V600E mutation may predict sensitivity to BRAF, MEK or ERK inhibitors.<br>Examples of clinical trials that may be appropriate for this patient are listed below. These trials were identified through a search of the trial website clinicaltrials.gov using keyword terms such as "BRAF", "V600", "MEK", "ERK", "HSP90", "vemurafenib", "dabrafenib", "LGX818", "regorafenib", "trametinib", "cobimetinib", "selumetinib", "MEK162", "NSCLC", "lung", "solid tumor", and/or "advanced cancer".

TITLE	PHASE	TARGETS	LOCATIONS	NCT ID
A Phase Ib/II, Multicenter, Open-label, Dose Escalation Study of LGX818 in Combination With MEK162 in Adult Patients With BRAF V600 - Dependent Advanced Solid Tumors	Phase 1/Phase 2	BRAF, MEK, CDK4, CDK6	Catalunya (Spain), MI (Italy), Madrid (Spain), Napoli (Italy), New South Wales (Australia), Paris (France), Quebec (Canada), Zuerich (Switzerland)	NCT01543698
Pilot Trial of Molecular Profiling and Targeted Therapy for Advanced Non-Small Cell Lung Cancer, Small Cell Lung Cancer, and Thymic Malignancies	Phase 2	MEK, AKT, CSF1R, EGFR, ERBB2, FLT3, KIT, PDGFRs, RET, VEGFRs	Maryland, Oregon	NCT01306045
Molecular Profiling-based Assignment of Cancer Therapy for Patients With Advanced Solid Tumors	Phase 2	MEK, MTOR, PARP, WEE1	Maryland	NCT01827384
A Phase I Trial of Vemurafenib in Combination With Cetuximab and Irinotecan in Patients With BRAF V600 Mutant Advanced Solid Malignancies	Phase 1	BRAF, EGFR, TOP1	Texas	NCT01787500
A Phase II Study of the BRAF Inhibitor Dabrafenib as a Single Agent and in Combination With the MEK Inhibitor Trametinib in Subjects With BRAF V600E Mutation Positive Metastatic (Stage IV) Non-small Cell Lung Cancer	Phase 2	BRAF, MEK	California, Colorado, Maryland, Massachusetts, Michigan, Missouri, New Hampshire, New York, Ohio, Pennsylvania, Washington, Multiple ex-US sites	NCT01336634

CLINICAL TRIALS TO CONSIDER (CONT.)

GENE	RATIONALE FOR POTENTIAL CLINICAL TRIALS
**ATM** Y264fs*12	Preclinical data suggest that loss or inactivation of ATM may lead to increased sensitivity to PARP inhibitors, as well as to DNA-PKcs inhibitors. Therefore, these classes of inhibitors may be a relevant approach for patients with ATM loss or inactivating mutations.<br><br>Examples of clinical trials that may be appropriate for this patient are listed below. These trials were identified through a search of the trial website clinicaltrials.gov using keyword terms such as "PARP", "olaparib", "rucaparib", "BMN 673", "ABT-888", "veliparib", "E7449", "DNA-PKcs", "lung", "NSCLC", "solid tumor", and/or "advanced cancer".

TITLE	PHASE	TARGETS	LOCATIONS	NCT ID
A Phase 1a/b, Multicenter, Open Label, Dose-Finding Study to Assess the Safety, Tolerability, Pharmacokinetics and Preliminary Efficacy of the Dual DNA-PK and TOR Kinase Inhibitor, CC-115, Administered Orally to Subjects With Advanced Solid Tumors, and Hematologic Malignancies.	Phase 1	DNA-PK, mTOR	California, Florida, New York, Tennessee, Texas, Barcelona (Spain), Koeln (Germany), Madrid (Spain), San Sebastian (Spain), Sevilla (Spain), Villejuif (France), Wuerzburg (Germany)	NCT01353625
A Randomised Phase II Trial of Olaparib Maintenance Versus Placebo Monotherapy in Patients With Chemosensitive Advanced Non-Small Cell Lung Cancer	Phase 2	PARP	Leicester (United Kingdom)	NCT01788332
A Randomized, Open Label, Multicenter, Phase 2 Trial Comparing Veliparib Plus Carboplatin and Paclitaxel Versus Investigators Choice of Standard Chemotherapy in Subjects Receiving First Cytotoxic Chemotherapy for Metastatic or Advanced Non-Squamous Non-Small Cell Lung Cancer (NSCLC) Who Are Current or Former Smokers.	Phase 2	PARP	Arkansas, Indiana, Michigan, Ohio, Tennessee	NCT02264990

APPENDIX

VARIANTS OF UNKNOWN SIGNIFICANCE

Note: One or more variants of unknown significance (VUS) were detected in this patient's tumor. These variants have not yet been adequately characterized in the scientific literature. We choose to include them here in the event that they become clinically meaningful in the future.

**APC**<br>T1160K	**CTNNB1**<br>N287S	**FANCA**<br>V340I	**FANCF**<br>D338N
**GNAS**<br>R147S	**MAP3K1**<br>S939C	**MLL3**<br>G4125S	**PIK3C2B**<br>R1037H
**PRKDC**<br>L933V,T920I			
